# Decoding the impact of nsSNP variants on BCL6 function through integrated computational analysis

**DOI:** 10.1016/j.crstbi.2026.100180

**Published:** 2026-02-02

**Authors:** Shaden M.H. Mubarak, Pardis Abdali Dehdezi, Amir Razavinia, Bahman Khalesi, Zahra Sadat Hashemi, Abolfazl Jahangiri, Mohammad Reza Rahbar, Mahdieh Mahboobi, Saeed Khalili

**Affiliations:** a Department of Clinical Laboratory Science, Faculty of Pharmacy, University of Kufa, Najaf, Iraq; b Independent Researcher, Slovakia; c Department of Genetics, Faculty of Advanced Science and Technology, Tehran Medical Sciences, Islamic Azad University, Tehran, Iran; d Department of Research and Production of Poultry Viral Vaccine, Razi Vaccine and Serum Research Institute, Agricultural Research, Education and Extension Organization, Karaj, 3197619751, Iran; e ATMP Department, Breast Cancer Research Center, Motamed Cancer Institute, ACECR, Tehran, Iran; f Applied Microbiology Research Center, Biomedicine Technologies Institute, Baqiyatallah University of Medical Sciences, Tehran, Iran; g Pharmaceutical Sciences Research Center, Shiraz University of Medical Sciences, Shiraz, Iran; h Department of Biology Sciences, Shahid Rajaee Teacher Training University, Tehran, Iran

**Keywords:** BCL6, Corepressors, DLBCL, Docking, MD simulations, nsSNPs

## Abstract

BCL6 plays significant roles in various cellular processes and malignancies such as diffuse large B-cell lymphoma. BCL6 performs its functions through binding of its BTB domain to different corepressors. Thus, analyzing the possible structural consequences of nsSNPs on the function of this domain would be imperative. To this end, we have selected the most deleterious SNPs of BCL6 based on various scoring algorithms. Then the selected mutations were modeled, analyzed for various physicochemical and stability properties, and used for molecular docking with the BCoR, NCoR, and SMRT. The obtained complexes were used for the calculation of binding energy and depiction of 2D interaction plots. The docked complexes were also subjected to Molecular Dynamics (MD) simulations to screen their behavior in physiological conditions. The BCL6 SNPs were filtered to 54 nsSNPs of the BTB domain. Using various tools, these nsSNPs were narrowed down to the Q113K, V105G, I78T, and I60T mutations based on their deleteriousness and stability scores. Docking analyses indicated that the exerted mutations mostly reduced the binding affinity, and the MD simulations showed the lower stability of the mutated BCL6 forms during the simulation. Given the attained results, it could be concluded that selected nsSNPs could lead to impaired BCL6 transcriptional repressive function due to loss of stability and binding affinity towards its corepressors. These observations can explain various biological or clinical differences in individuals carrying these SNPs and help with the rational design of novel personalized therapeutics. The results found by computer simulations are suggesting new experiments that need to be done in the future to prove that they are biologically and clinically applicable.

## Highlights

• Four deleterious nsSNPs (Q113K, V105G, I78T, I60T) were identified in the BCL6 BTB domain.
• These mutations reduce BCL6 binding affinity to corepressors BCoR, NCoR, and SMRT.
• MD simulations show increased instability and flexibility in mutant BCL6-corepressor complexes.
• Mutations cause slight changes in phosphorylation, glycosylation, and ubiquitination sites.
• BCL6 nsSNPs may disrupt transcriptional repression and contribute to lymphomagenesis.

## Graphical abstract

**Figure:**
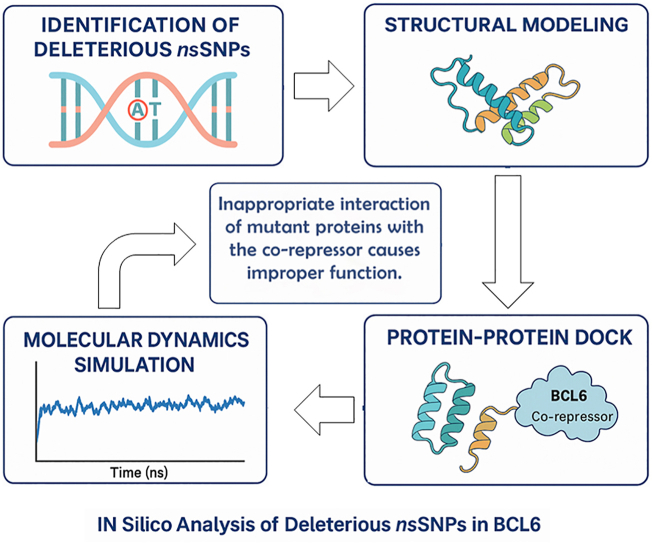

## 1 Introduction

The B-cell lymphoma 6 (BCL6) is a transcriptional repressor. It plays a pivotal role in the humoral immune responses. Regulation of the germinal center (GC) formation and activities in lymphoid tissues is among the BCL6 functions (Basso et al., 2010). This transcriptional repressor is predominantly expressed in GC B-cells. BCL6 is involved in silencing various genes, which are essential for cellular processes such as B-cell differentiation, proliferation, and survival. Cell cycle arrest and programmed cell death (apoptosis) could be among the consequences of BCL6-mediated gene silencing. BCL6 contains two key catalytic domains, which include the BTB/POZ domain (N-terminus) and zinc finger motifs (C-terminus). The repressive capabilities of the BCL6 highly rely on these domains. The BTB/POZ domain promotes the homodimerization of BCL6. It also recruits specific BCL6 co-repressors, including the BCL6-interacting corepressor (BCoR), nuclear receptor corepressor (NCoR), and silencing mediator of retinoic acid and thyroid hormone receptor (SMRT). Complexes of BCL6 with these co-repressors bear histone deacetylase activity, which could lead to chromatin condensation and inhibition of gene expression (Bonchuk et al., 2023; Liongue et al., 2024). The BTB/POZ domain acts as a scaffold for the assembly of multi-protein repressive units. These units are required for efficient transcriptional silencing of the target genes. Thus, the BTB/POZ-mediated protein-protein interactions are essential for the efficient functionality of BCL6. On the other hand, the zinc finger motifs of BCL6 are required for its DNA-binding property. These motifs enable the BCL6 to target genes involved in various processes such as cell cycle progression, immune regulation, and DNA repair (McLachlan et al., 2022). The BCL6 is a master gene regulator due to its dual-domain architecture. It can coordinate the fine balance of B-cell maturation, which is required for a successful adaptive immunological response. Dysregulation of BCL6 often happens via chromosomal translocations or somatic mutations. Deregulated BCL6 expression is a defining feature of lymphoid malignancies such as diffuse large B-cell lymphoma (DLBCL) (Zhou et al., 2018). Aberrant expression of BCL6 in DLBCL can result in lymphomagenesis by disrupting normal gene regulation, stimulating uncontrolled B-cell proliferation, and promoting cell survival. Thus, BCL6 can act as a physiological regulator and a potential oncogene (Cardenas et al., 2017). Getting a better grasp of the mechanisms behind BCL6 dysregulation seems crucial to underscore its role in both maintaining immune homeostasis and contributing to malignancy.

Although the somatic mutations and translocations of the BCL6 gene have been extensively investigated, the effects of germline single-nucleotide polymorphisms (SNPs) (especially non-synonymous SNPs (nsSNPs)) on its function remain to be explored. NsSNPs are the polymorphisms that cause amino acid substitutions in the protein sequence. These polymorphisms are the most common genetic variants in the human genome and can lead to drastically altered structure, stability, or interactions of proteins (Yates et al., 2013). Deleterious nsSNPs might impair critical domains of BCL6 (hindering the formation of repressive complexes and/or reducing DNA binding specificity) and disrupt its transcriptional repression. Increased susceptibility to diseases like DLBCL or altered immune functions could be among the potential consequences of impaired functions of BCL6. Given the central role of BCL6 in immunity and cancer, it is important to understand how these genetic variants would modify its behavior in both normal biological processes and pathological states. Moreover, elucidating the effects of nsSNPs could bring about significant implications for personalized medicine and tailoring diagnostic and therapeutic approaches to individual genetic profiles (upon identifying nsSNPs that influence disease risk or treatment efficacy). The value of studying nsSNPs becomes more evident given their potential to customize medical interventions, which could bridge the gap between genetic variation and clinical outcomes. However, over 11000 SNPs have been identified in BCL6. Empirical investigation of the SNPs would require immense resources. This challenge indicates the limitations of conventional experimental approaches and highlights the necessity of alternative strategies.

In silico methods have recently emerged as amenable alternatives to empirical methods that can address their limitations (Khalili, 2017; Mohammadpour et al., 2015; Jahangiri, 2012). These methods could exploit computational tools to predict the functional consequences of nsSNPs (Yazar et al., 2021). They can analyze the effect of SNPs on protein stability, folding, and molecular interactions. These data could help to prioritize nsSNPs for future experimental analyses (Wang et al., 2013). Bioinformatics tools can model the effect of nsSNPs on BCL6 co-repressor binding or DNA recognition and provide novel insights into their pathogenicity. In the present study, we aim to find and investigate deleterious nsSNPs in BCL6. Moreover, their effects on BCL6 structure and interactions with BCoR, NCoR, and SMRT co-repressors would be predicted. The molecular mechanisms underlying BCL6-related pathologies could be explained using the obtained results. Furthermore, these findings could delineate the clinical relevance of these nsSNPs and potentially help to develop targeted therapies or diagnostic markers. Understanding how genetic variations can modulate the function of BCL6 could be deemed an important step toward the elucidation of the interplay between genetics, protein activity, and disease.

## 2 Methodology

The current work has used a holistic approach of in silico (computer-based) tools to analyze the BCL6 protein. The main focus was the detection and characterization of non-synonymous single-nucleotide polymorphisms (nsSNPs) and their pragmatic consequences. The complete process involved downloading data from public databases, multi-tool computational predictions, and advanced structural and dynamic modeling.

### 2.1 Data acquisition and SNP identification

The BCL6 protein's SNP data were found in the NCBI dbSNP (https://www.ncbi.nlm.nih.gov/snp/) and the Ensembl genome browser (https://www.ensembl.org/) databases. These sources show us global minor allele frequencies, residue changes, and location on the genome with complete details. In all, the cases were caused by nonsynonymous single-nucleotide polymorphisms (nsSNP), and cases were successfully evaluated, thus making them the foundation for more thorough deleteriousness predictions.

### 2.2 Computational prediction of deleterious nsSNPs

Robust prediction of deleterious nsSNPs in BCL6 was performed using widely used and validated computational tools such as Meta-SNP (https://snps.biofold.org/meta-snp/) (Capriotti et al., 2013), SIFT (https://sift.bii.a-star.edu.sg/) (Kumar et al., 2009), PolyPhen-2 (http://genetics.bwh.harvard.edu/pph2/) (Kumar et al., 2009), PhD-SNP (https://snps.biofold.org/phd-snp/phdsnp.html) (Calabrese et al., 2008), and SNAP (http://snap.genomics.org.cn/) (Li et al., 2007). The reason for using these tools is that they apply complementary methodologies (sequence homology, structural impact, and machine learning models) and are commonly used in high-confidence variant filtering pipelines. to improve consensus reliability Meta-SNP integrates multiple predictors, evolutionary conservation and amino acid substitution tolerance evaluates by SIFT, structural and comparative features are evaluated by PolyPhen-2, PhD-SNP and SNAP rely on machine learning and neural networks trained on annotated variant datasets. None of these tools were specifically developed for BCL6, but they have shown high accuracy (ranging between 70 and 85%) across a variety of proteins, and the combined use of these tools reduces false positives and improves predictive power. Default confidence thresholds were used for all tools; for high specificity, only cases predicted to be harmful in all tools were selected for subsequent analyses. Other predictors were also considered for this study but were excluded due to a lack of specific interpretability for BCL6 functional domains or limited accessibility.

### 2.3 Clinical and functional annotation

The ClinVar (Landrum et al., 2016) (https://www.ncbi.nlm.nih.gov/clinvar/) database was used to deepen the clinical significance of the SNPs, which aggregates genomic variation data and its correlation with human health. Besides, linkage with disease phenotypes was sought through the GWAS Catalog (MacArthur et al., 2017) (https://www.ebi.ac.uk/gwas/). For a finer functional annotation, tools such as the Ensembl Variant Effect Predictor (VEP) (McLaren et al., 2016) (https://www.ensembl.org/vep) and ANNOVAR (Wang et al., 2010) (https://wannovar.wglab.org/index.php) were employed. These genetic studies determined whether the SNPs were found in coding or regulatory regions, and on the other hand, the mutations were classified as missense, nonsense, or silent.

### 2.4 SNP impact on BCL6 gene expression

Two web servers, namely RegulomeDB (Boyle et al., 2012) (https://www.regulomedb.org/regulome-search/) and GTEx Portal (Stanfill et al., 2021) (https://www.gtexportal.org/home/), were used to test the influence of SNPs on expression alterations in BCl6 protein and some other relevant genes in different tissues.

### 2.5 Assessment of protein stability and structural integrity

The conservation of protein was validated by I-Mutant 2.0 (Capriotti et al., 2005) (http://folding.biofold.org/i-mutant/i-mu-tan-t2.0.html), a software which gives predictions using a support vector machine algorithm based on the changes induced by amino acid substitutions under different sets of physical conditions such as either at the standard temperature (25 °C) or the physiological temperature (37 °C) and the sequence of the protein as input. Moreover, the ProtParam (Garg et al., 2016) (https://web.expasy.org/protparam/) was used as another tool for the complementary analysis of the physicochemical properties of the protein. Residues that are conserved over time at the evolutionary level were identified by the DeepREx-WS (Manfredi et al., 2021) (https://deeprex.biocomp.unibo.it/) web server and the CLC Genomics workbench (version 25.0.1) program. This analysis made it possible to identify highly conserved residues that correspond to the deleterious nsSNPs.

### 2.6 Secondary structure prediction and 3D structural modeling

Given that the full-length crystal or NMR structure of BCL6 is not available, this study focused on the BTB/POZ domain of the protein (N-terminal region, residues 1–129). This region plays a critical role in binding to corepressors and is identified in the Protein Data Bank with the ID 6XMX. Given its importance in protein-protein interactions, it was selected as a target for variant modeling and interaction analysis. Variants outside this region were excluded due to the lack of reliable structural models. PSIPRED (McGuffin et al., 2000) (https://bio.tools/psipred) was used to make secondary structure predictions, which include the α-helices, β-strands, and coils, among others. The pCysMod (Li et al., 2021) (http://pcysmod.omicsbio.info/) program was used to predict alternative cysteine modifications for the mutant stability analysis. Then, Molegro software (version 6.0) (Kusumaningrum et al., 2014) was used to create a 3D model structure of mutant BCL6 proteins of the wild-type protein, which was next checked for structural variation using the TM-align (Zhang et al., 2005) (https://zhanggroup.org/TM-align/) program. This server calculated the TM-scores and RMSD values. The CLC genomic workbench (Matvienko, 2015) served as a platform for the study of the resulting structures of selected mutants that exhibit significant deviations. The quality of the predicted 3D model was evaluated by the QMEAN (Benkert et al., 2009) (https://swissmodel.expasy.org/qmean/) tool.

### 2.7 Protein-protein interaction and docking analysis

To scrutinize the influence of harmful mutations on protein interactions, docking studies were conducted with HADDOCK 2.4 (Honorato et al., 2024) (https://rascar.science.uu.nl/haddock2.4/). We have used the default settings of the server for docking analyses. The re-docking of native BCL6 with its main native co-repressors (BCoR, NCoR, and SMRT) was also done using this server. The native structure of BCL6-BCoR was obtained from PDB ID: 3BIM, the BCL6-NCoR was obtained from PDB ID: 6XYX, and the BCL6-SMRT was obtained from PDB ID: 1R2B. In this regard the Interaction interfaces between the BCL6 mutants and their main co-repressors were displayed with LigPlot (version 2.2.9), and the values of binding affinities were measured quantitatively by the PRODIGY server using the default settings (Xue et al., 2016) (https://rascar.science.uu.nl/prodigy/).

### 2.8 Molecular dynamics simulation

Molecular dynamics (MD) simulations were conducted using the WebGro (Paz et al., 2004) (https://simlab.uams.edu/) server over simulation times of 200 ns. Analyses of RMSD, RMSF, radius of gyration, and hydrogen bond patterns allowed for a complete understanding of the stable form of the proteins under simulated physiological conditions by using the above method.

### 2.9 Post-translational modification analysis

To ensure that the mutations indicated have actual impacts, a full review of the PTMs done in the BCL6 protein has been made. In GPS-MSP 1.0 (Hossain et al., 2020) (http://msp.biocuckoo.org/online.php), the predicted methylation sites in the BCL6 protein were discovered. The phosphorylation sites on serine, threonine, and tyrosine residues were predicted using NetPhos 3.1 (Sivakumar et al., 2025) (https://services.healthtech.dtu.dk/services/NetPhos-3.1/) and GPS 6.0 (Das et al., 2017) (https://gps.biocuckoo.cn/index.php). The task of checking on the possibility of ubiquitination was carried out by use of the RUBI (Iqbal et al., 2024) (https://old.protein.bio.unipd.it/rubi) and GPS-Uber (Iqbal et al., 2024) (https://gpsuber.biocuckoo.cn/) tools, which were concentrating specifically on lysine residues. The RUBI has been employing an equal cut-off criterion to ensure lysine ubiquitination prediction. Furthermore, to make the O-linked glycosylation sites prediction in the BCL6 protein, the NetOglyc 4.0 (Khrustalev et al., 2012) (https://services.healthtech.dtu.dk/services/NetOGlyc-4.0/) was used. Data related to glycosylation patterns between the wild-type and mutant forms of the protein helped to reveal the possible functional changes linked to the mutations.

### 2.10 Gene-gene and domain interaction analyses

The search was also carried out in gene-gene and domain interaction, which was accomplished using tools such as GeneMANIA (Zuberi et al., 2013) (https://genemania.org/) and STRING (Snel et al., 2000) (https://string-db.org/). A network of interaction data was built using co-expression, pathway sharing, and experimental evidence as the basis. Other functions, such as domain and motif analyses, were performed using Pfam (Finn et al., 2006) (http://pfam.xfam.org/) to decide whether nsSNPs modified the functional domains like the BTB/POZ domain, which is important in protein-protein interactions and DNA binding.

## 3 Results

### 3.1 Data acquisition and SNP identification

11,697 SNPs were identified to be associated with the BCL-6 protein using the SNP database. Hence, in total, there were 577 of these cases caused by nonsynonymous single-nucleotide polymorphisms (nsSNP). These SNPs, which are associated with amino acid changes within the BCL6 protein sequence, were used as the basis of further deleteriousness assessments.

### 3.2 Computational prediction of deleterious nsSNPs

The nsSNPs were analyzed by Meta-SNP, SIFT, PolyPhen-2, PhD-SNP, and SNAP tools. Only those were selected that were reported as pathogenic in all the aforementioned tools. Due to the lack of a complete three-dimensional structure of the BCL-6 protein in databases, the structure of this protein in the PDB databank under the ID of 6XMX was selected for further analysis. This structure includes amino acids 1 to 129, given that there is no empirical 3D structure beyond residue 129, variants outside the BTB domain were excluded. Our analysis focused on the BTB region because it is critical for protein function and mediates binding of the protein to corepressors, and is also structurally known (PDB: 6XMX). Analysis of other domains of this protein could be performed in the future as structural data become available. Table 1 lists the rsIDs of the SNPs in this region and the sequence of the amino acid changes in these rsIDs. The nsSNP selection criteria at each stage are shown in Table 2.

**Table 1:** List of predicted deleterious nsSNPs in the BCL6 protein.

No	SNP	Mutation	No	SNP	Mutation	No	SNP	Mutation
1	rs1719151122	C8S	19	rs2108471511	A52G	37	rs867240773	R98W
2	rs1204164230	F11S	20	rs1415288002	F57L	38	rs377166099	G100V
3	rs2108471713	T12I	21	rs752436963	S59G	39	rs1719058118	M103V
4	rs772289458	T12P	22	rs955595035	I60T	40	rs200844445	V105G
5	rs772289458	T12S	23	rs1327801709	F61L	41	rs201271781	M106V
6	rs2108471698	R13C	24	rs1719065575	T62I	42	rs1719056630	T108M
7	rs2108471694	R13H	25	rs767073067	N68K	43	rs1719056277	M110V
8	rs2108471687	A15V	26	rs911170380	N68S	44	rs1719055545	Q113H
9	rs2108471677	D17E	27	rs1202329522	N73D	45	rs754593601	Q113K
10	rs2108471658	N23I	28	rs1719063001	L74I	46	rs1719055352	E115Q
11	rs746411980	R24C	29	rs1579816551	D75Y	47	rs1218169171	H116R
12	rs752260228	R24H	30	rs774593110	I78T	48	rs866452181	V117F
13	rs779268890	R26G	31	rs763472780	P80S	49	rs751185760	D119N
14	rs779268890	R26W	32	rs1719061731	G82E	50	rs1719053867	T120S
15	rs11545363	R28G	33	rs148348997	F89L	51	rs551620719	R122W
16	rs2108471555	K47T	34	rs781532443	R94W	52	rs1579816201	F124V
17	rs200263685	T48M	35	rs867240773	R98C	53	rs765718723	K126R
18	rs2108471523	L50H	36	rs1391779185	R98P	54	rs1368125173	A127P

**Table 2:** nsSNP filtering and selection.

Step	Description	SNP Count
1	Total BCL6 SNPs from dbSNP/Ensembl	11,697
2	Filtered to nsSNPs	577
3	Predicted deleterious by 5 tools	54
4	Located within residues 1–129 (BTB domain)	54
5	Prioritized based on stability, conservation, and structure	4 (I60T, I78T, V105G, Q113K)

### 3.3 Clinical and functional annotation

During the analysis with the ClinVar database, designed to study genomic variation, and the GWAS Catalog (designed to study the relationship between SNPs and phenotype), it was found that the selected nsSNPs had no information in these two databases. On the other hand, VEP and ANNOVAR tools were utilized for adequate functional annotation. The use of the Variant Effect Predictor (VEP) web server for BCL6 mutation examination revealed the presence of missense variants, such as rs774593110, rs200844445, rs754593601, and rs955595035, which could significantly affect the function of the protein. The above mutations, for example, all resulted in amino acid substitutions that changed the protein's structure and function. A deeper investigation of the BCL6 gene using WANNOVAR exposed several missense variants that were highly significant. Particularly, rs774593110, rs200844445, rs754593601, and rs955595035 were the ones with higher function-affecting potential. It's important to mention that both the VEP and ANNOVAR results confirmed that the target nsSNPs are near or inside the functional domains of BCL6. Specifically, these alterations were more likely to disrupt the domains that contribute to DNA binding or protein-protein interactions.

### 3.4 SNP impact on BCL6 gene expression

There was no information about the investigated SNPs on the GTEx Portal web server. A few SNPs have been removed due to the lack of information, and some SNPs adversely affected the gene expression while we studied them on the RegulomeDB. The rs774593110, rs200844445, rs754593601, and rs955595035 were also among the items that were determined to bring about the changes in the gene expression in different tissues.

### 3.5 Assessment of protein stability and structural integrity

**Fig. 1:**
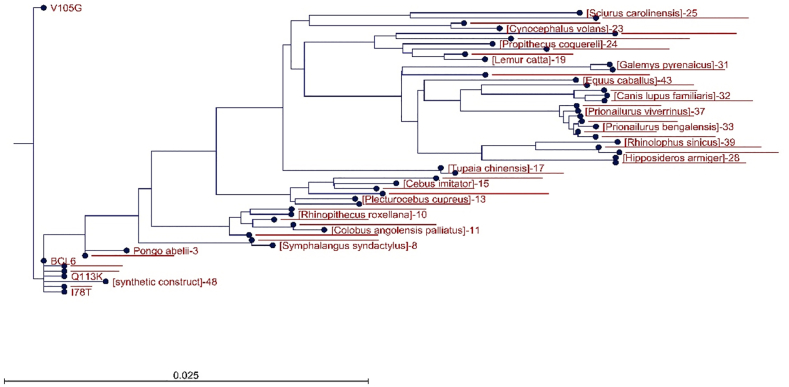
phylogenetic tree of mutation in BCL6.

The stability of the nsSNPs in the protein structure was assessed using the I-Mutant 2.0 tool. From the results obtained from the I-Mutant web server, mutations that increase protein stability and those that show the highest degree of instability were selected to continue the process. The rs754593601 mutation was shown to increase stability, and the rs200844445, rs774593110, and rs955595035 mutations were shown to decrease stability. The amino acid changes of these mutations were Q113K, V105G, I78T, and I60T, respectively. Additional analyses utilizing the ProtParam server provided a further view of the physicochemical features of the protein. After reviewing the solvent-accessibility analysis, we found that none of the four mutations significantly increased the overall BCL6 surface exposure. The wild-type protein exposure was calculated to be about 61%. The mutant proteins were not that far from the wild-type, as they all represent from 59% to 61% of the reference (I60T and Q113K at 60%; I78T and V105G at 59%). Using the CLC Genomic Workbench software and applying 50 homologous sequences, the percentage of conserved amino acid residues was more than 70%, and a phylogenetic tree was drawn. This analysis showed that the V105G mutation causes the greatest amount of change in the structure of BCL6 compared to the rest of the mutations. (Fig. 1). Hazardous nsSNPs thus found were further analyzed with conserved residues.

### 3.6 Secondary structure prediction and 3D structural modeling

Because the 3D structure of the BCL6 protein beyond residue 129 was not available, the modeling performed in this study was limited to the available residues. According to the PSIPRED secondary-structure predictions, all the four point mutations do not alter the local structure of BCL6: residue 60 is still in an α-helix in the wild type and I60T; residue 78 is a β-strand in the wild type and I78T; residue 105 is still a part of the coil next to a helix in both wild type and V105G; and residue 113 is still the part of a solvent-exposed loop in both wild type and Q113K. The pCysMod web server was used to evaluate whether there were any cysteine modifications in the protein structure. It was found that there were no cysteine modifications in the protein sequences by this web server. The potential 3D structures of the mutant sequence were prepared using the Molegro software. These mutated BCL6 protein structures, which had the highest conformity to the original structure, were used for the following analyses. Using the TM-align web server, TM scores and RMSD values were calculated between the wild type and the mutated structures. The TM score of all structures was 0.992, and the RMSD values were calculated to be 0.0. It is evident that despite the exerted mutations, both compared structures shared the same fold. QMEAN analysis for wild-type BCL6 and I60T, I78T, V105G, and Q113K mutations resulted in the QMEAN score of −1.06, −1.08, −1.10, −1.14, and −1.24, respectively. The QMEAN scores were close to zero, which is preferable for a more accurate and reliable model. Therefore, the mutation of the protein did not significantly change its structure. The QMEAN score for the mutated models is higher than that of the wild-type protein, demonstrating that the prediction of the mutant structure is less unstable than the wild-type structure in terms of structural quality. Although AlphaFold could be used to predict the overall BTB fold, its global re-prediction approach is less suitable than the employed crystal-template-based local refinement for assessing the structural impact of single-point mutations in this domain (Buel et al., 2022) (Buel and Walters, 2022).

### 3.7 Protein-protein interaction and docking analysis

**Fig. 2:**
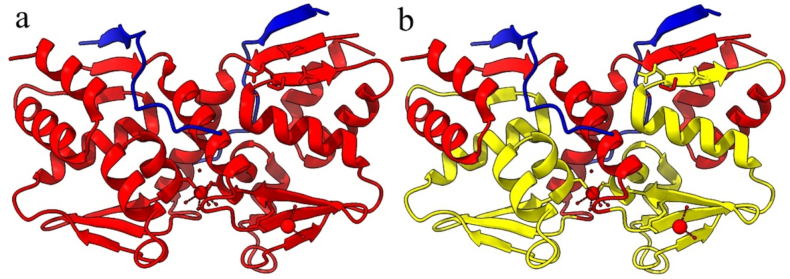
The structure of the BCL6 (spanning amino acids 1–129) in interaction with the NCOR corepressor. On the left (a) the BCL6 dimer is in red, while the NCOR corepressors are in blue. On the right (b) the same coloring is applied, while the BTB domain (spanning amino acids 32–99) is depicted in yellow.

**Fig. 3:**
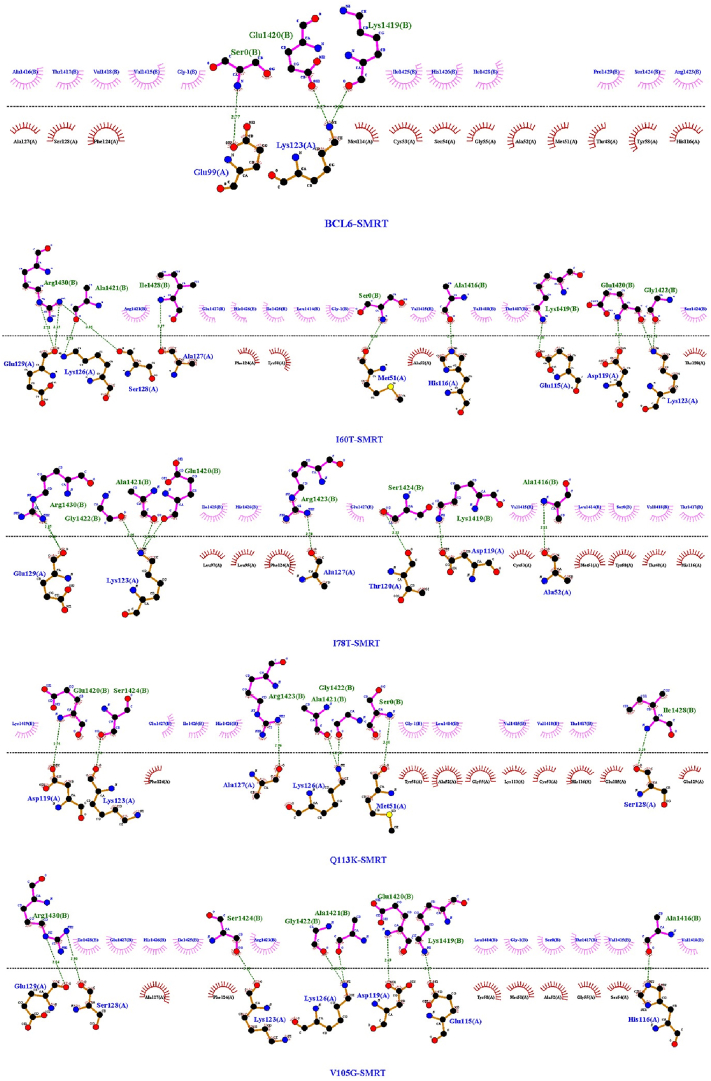
BCL6 and mutations interaction with SMRT.

**Fig. 4:**
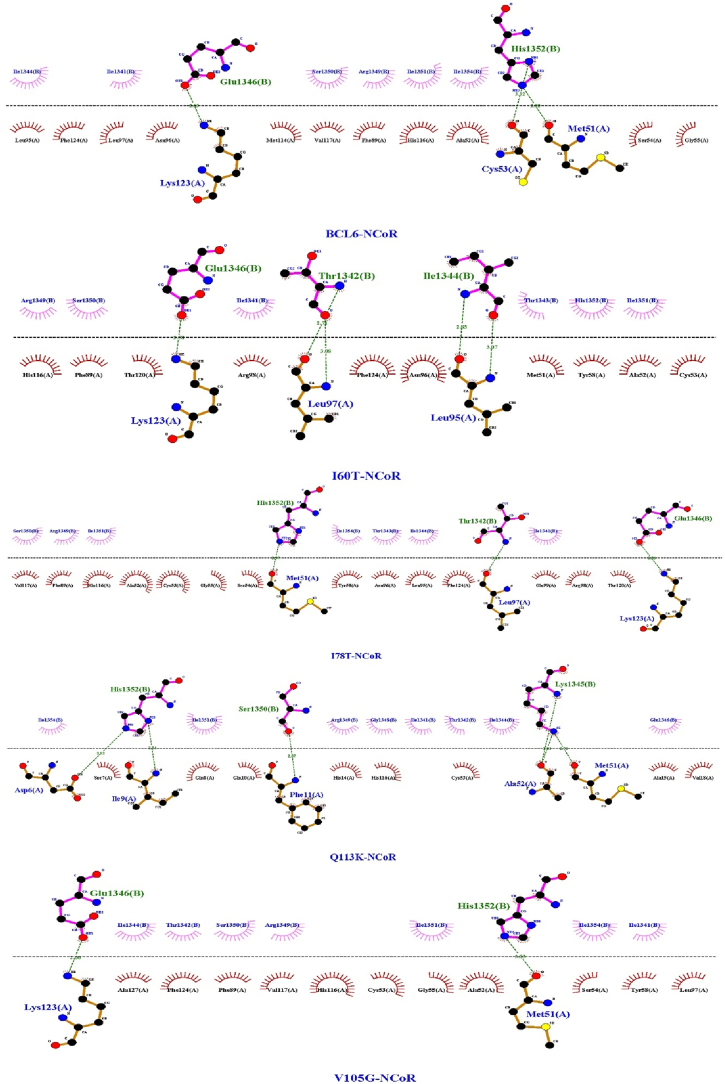
BCL6 and mutations interaction with NCoR.

**Fig. 5:**
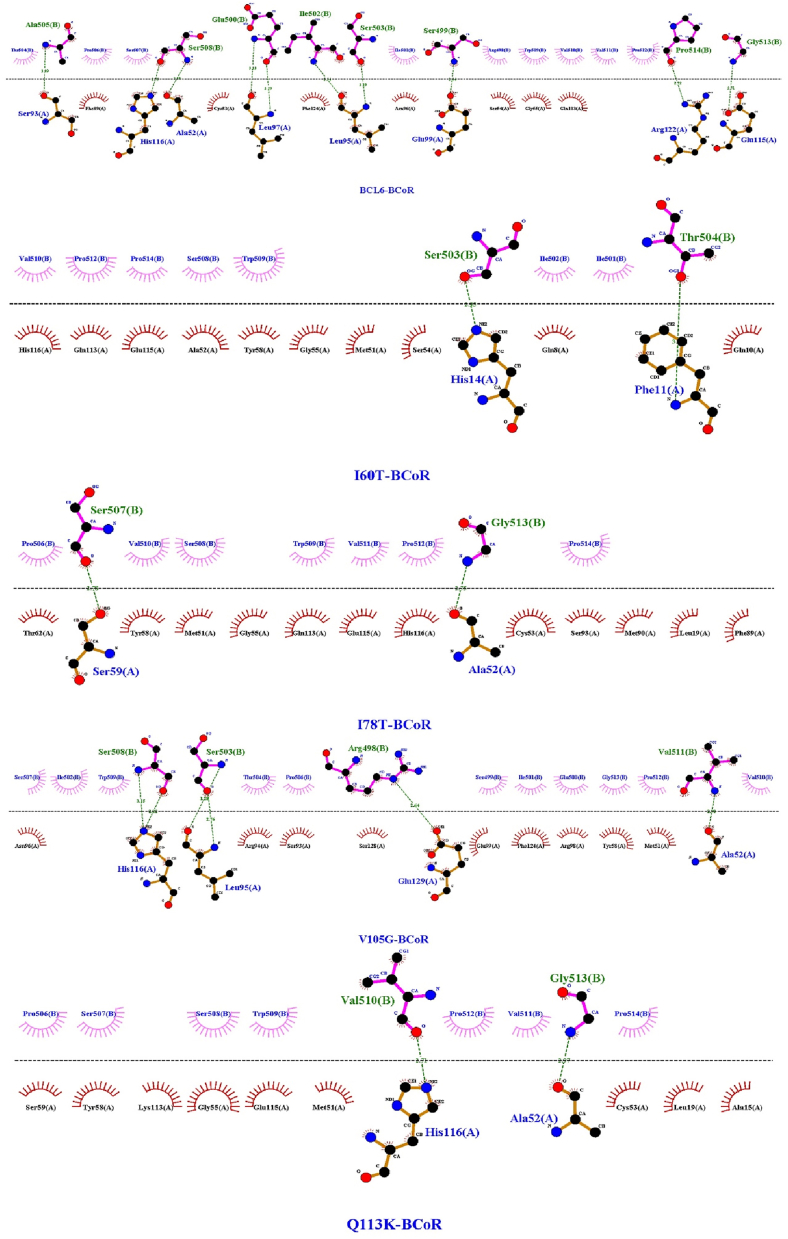
BCL6 and mutations interaction with BCoR.

Molecular docking was achieved through the HADDOCK 2.4 web server to examine the interactions between both the BCL6 and its mutant forms on the wild-type co-repressors (BCoR, NCoR, and SMRT) (Fig. 2). The results of which are shown in Table 3 (more detailed scores are presented in supplementary Table). Visualizations of interaction interfaces were done with LigPlot (Fig. 3, Fig. 4, Fig. 5), and the binding affinities were estimated quantitatively by the PRODIGY server. Docking positions were determined using 3D structures of BCL6 binding and corepressors, available in the PDB (BCL6-BCoR PDB ID: 3BIM, BCL6-NCoR PDB ID: 6XYX, BCL6-SMRT PDB ID: 1R2B). For each docking test performed by the HADDOCK 2.4 server, we generated multiple clusters of docking solutions. The HADDOCK score and the lowest energy conformations were the criteria for selecting the superior cluster. To evaluate docking modes, compatibility among clusters, cluster size, buried surface area, alignment of interacting residues using LigPlot+, binding affinity estimation using PRODIGY were examined to avoid selection bias based on HADDOCK score.

**Table 3:** Docking and binding affinity results of BCL6 and its mutant proteins on the co-repressors (BCoR, NCoR, and SMRT). The Wild-type ΔG was calculated by re-docking of BCL6 with its wild type BCoR, NCoR, and SMRT co-repressors (BCL6-BCoR complex was obtained from PDB ID: 3BIM; BCL6-NCoR complex was obtained from PDB ID: 6XYX; BCL6-SMRT complex was obtained from PDB ID: 1R2B).

Complex	ΔG Wild-type (kcal/mol)	ΔG Mutant (kcal/mol)	ΔΔG (WT – Mutant) (kcal/mol)
BCL6–SMRT (I60T)	−8.6	−7.9	−0.7
BCL6–SMRT (I78T)		−7.5	−1.1
BCL6–SMRT (V105G)		−8.7	+0.1
BCL6–SMRT (Q113K)		−8.5	−0.1
BCL6–NCoR (I60T)	−8.6	−8.5	−0.1
BCL6–NCoR (I78T)		−8.4	−0.2
BCL6–NCoR (V105G)		−6.9	−0.7
BCL6–NCoR (Q113K)		−8.1	−0.4
BCL6–BCoR (I60T)	−10.6	−7.3	−2.3
BCL6–BCoR (I78T)		−7.8	−2.8
BCL6–BCoR (V105G)		−6.3	−4.3
BCL6–BCoR (Q113K)		−7.0	−3.6

### 3.8 Molecular dynamics simulation

**Fig. 6:**
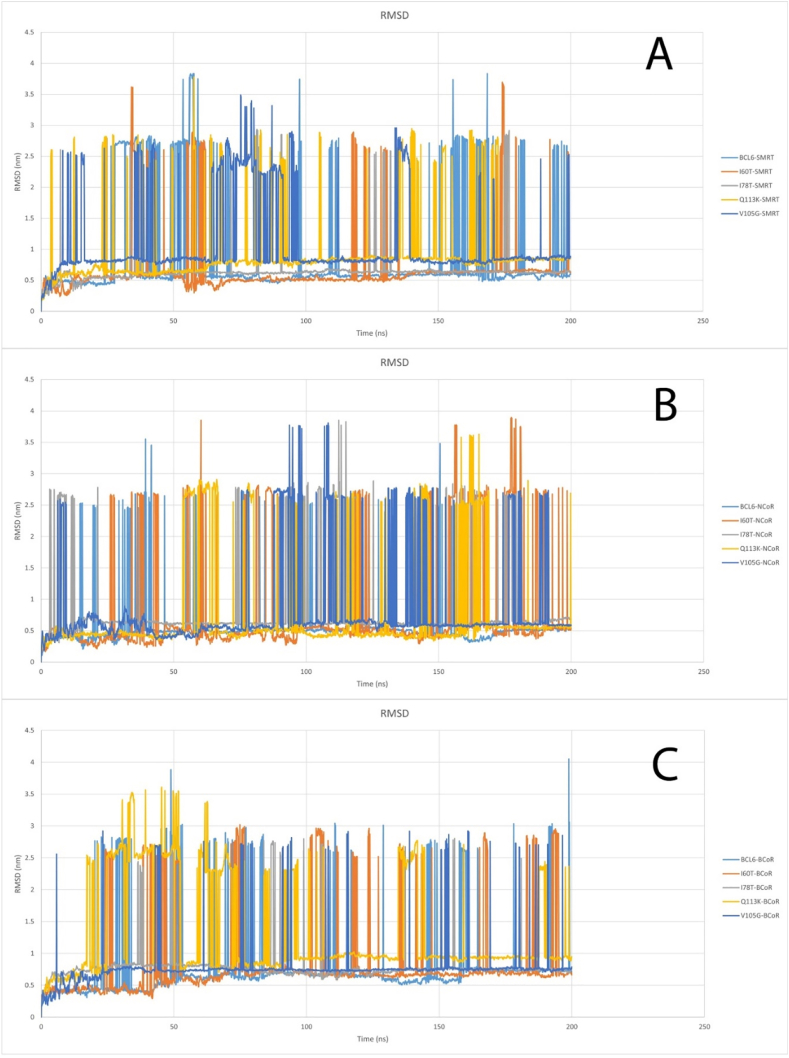
RMSD plot for BCL6 protein and all mutations and SMRT (A), RMSD plot for BCL6 protein and all mutations and NCoR (B), RMSD plot for BCL6 protein and all mutations and BCoR (C).

Molecular dynamics (MD) simulations were carried out utilizing the WebGro server for 200 ns. Lower RMSD values often suggest that the proteins are less unstable and are closer to the reference structure during the simulation. On the contrary, higher RMSD values inform us about the proteins that are less stable and that have larger deviations from the reference structure. Based on the results obtained from MD simulations for SMRT interactions, a lot of noise was observed in all structures, but in general, the V105G and Q113K mutations showed more instability than the other structures. In the case of BCoR, the Q113K mutation showed the highest degree of instability compared to the other structures, with a high level of noise observed in all structures. Based on the results obtained from MD simulations for NCoR interactions, the wild-type BCL6 protein was slightly more stable than all the mutations. All structures showed the same conditions and had a lot of noise. (Fig. 6). The settings used for the molecular dynamics simulation are given in Table 4.

**Table 4:** Settings used for MD simulation.

Name of setting	Selected setting
Forcefield	GROMOS96 43a1
Water model	SPC
Box Type	Triclinic
Salt Type	0.15 M NaCl
Integrator	Steepest Descent (steps:5000)
Equilibration Type	NVT/NPT
Temperature (K)	298
Pressure (bar)	1
MD integrator	Leap-Frog
Simulation time (ns)	200
Approximate number of frame per simulation	1000

**Fig. 7:**
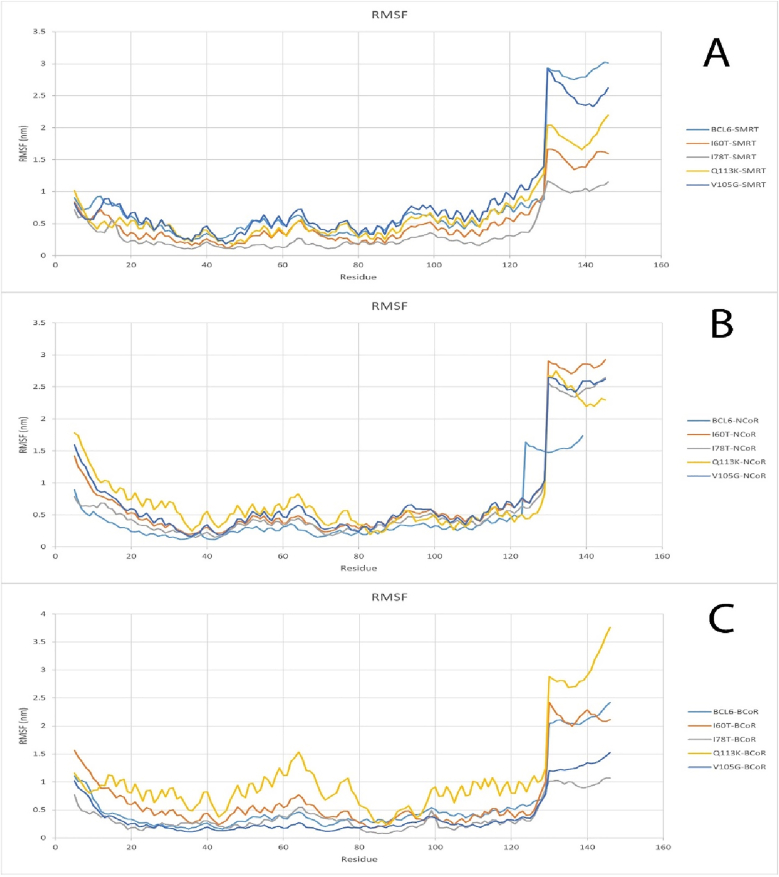
RMSF plot for BCL6 protein and all mutations and SMRT (A), RMSF plot for BCL6 protein and all mutations and NCoR (B), RMSF plot for BCL6 protein and all mutations and BCoR (C).

RMSF graph represents the difference of each residue from the protein backbone from its mean position. The bigger the RMSF value, the more flexibility and movement it shows. A lower RMSF value signifies great stability and less movement for the residue. For SMRT corepressor, The V105G mutation showed a function similar to the wild-type protein, and in the remaining cases, a lower RMSF value was evaluated. In the BCoR interactions result, Mutations Q113K and I60T showed a higher RMSF value on average than the others, the rest had almost similar performance. In the NCoR complex RMSF graph, All structures had almost the same function, but after amino acid 120, all mutations showed a higher RMSF value than the wild-type protein (Fig. 7).

**Fig. 8:**
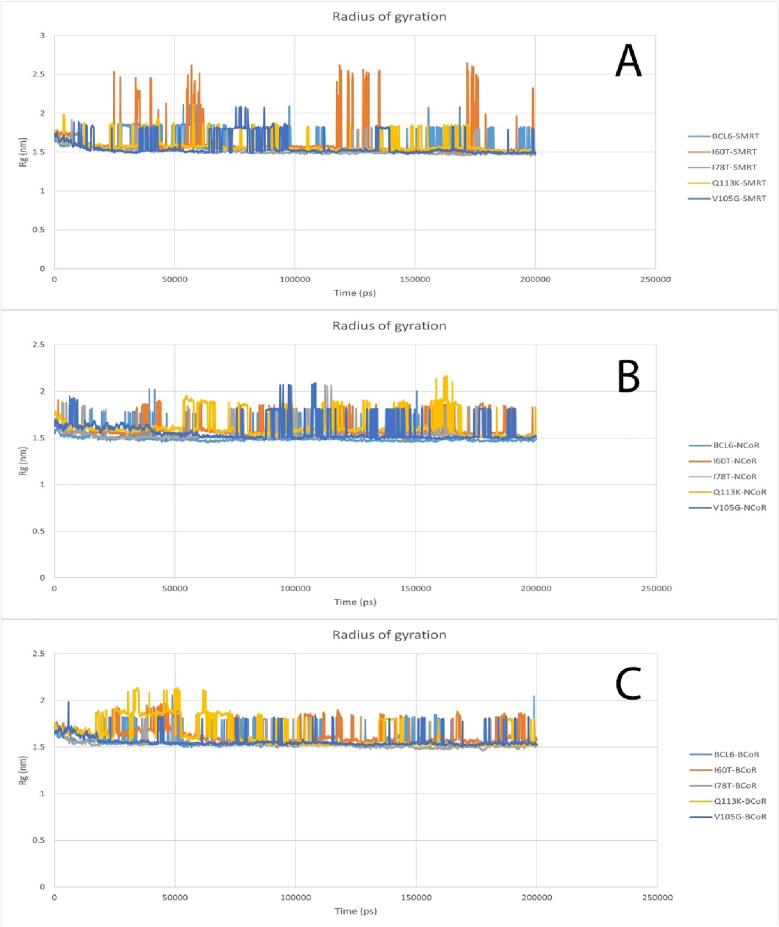
Radius of gyration plot for BCL6 protein and all mutations and SMRT (A), Radius of gyration plot for BCL6 protein and all mutations and NCoR (B), Radius of gyration plot for BCL6 protein and all mutations and BCoR (C).

The radius of gyration is a measure of the compactness of a molecule, with a higher value indicating a larger or more spread-out molecule. In all structures related to all three corepressors, the same performance with high noise was observed (Fig. 8).

**Fig. 9:**
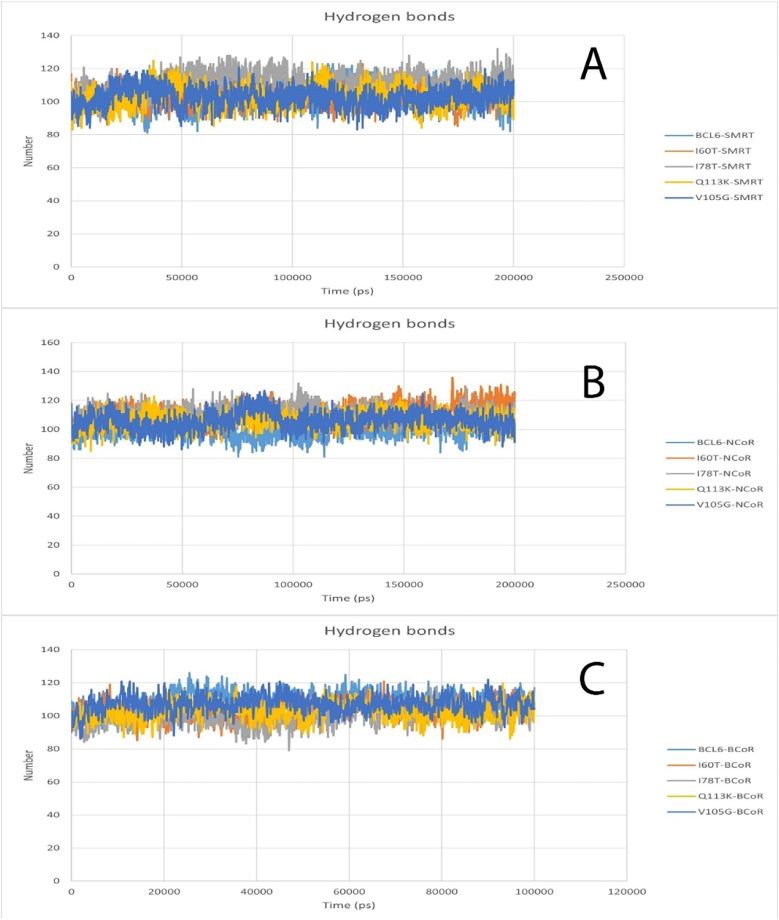
Hydrogen bond plot for BCL6 protein and all mutations and SMRT (A), Hydrogen bond plot for BCL6 protein and all mutations and NCoR (B), Hydrogen bond plot for BCL6 protein and all mutations and BCoR (C).

The hydrogen bond plot shows the hydrogen bonds formed over time during an MD simulation. The results obtained for all structures in all 3 cases, BCoR, NCoR, and SMRT, are the same and equal to 100 (Fig. 9).

Given that replication was not performed for MD tests, it was not possible to perform statistical analyses, so minor fluctuations were interpreted with caution. It is suggested that future studies replicate simulations and statistical tests for a more thorough evaluation.

### 3.9 Post-translational modification analysis

The expected phosphorylation scores of the BCL6 protein at ten different sites in the case of the wild type and the mutant variants (I60T, I78T, Q113K, and V105G) were predicted by GPS 6.0 kinase-specific predictions. In general, the phosphorylation pattern has only been moderately changed in all of the variants. As S16 (CAMK) and S271 (CK1) are the highly scored and, therefore, most likely the functional sites in the wild type, these two sites have not experienced any variations in the score. However, some slight mutations have occurred as a result of which specific phosphorylation scores have undergone minor fluctuations, such as T92, one more target site of AGC and CK1 kinases. In the I78T variant, the CK1-mediated phosphorylation score for T92 increases from 0.0950 to 0.0992, and this means the site has only slightly improved phosphorylation potential, while the AGC association has dropped. Q113K mutation has resulted in the T92 score of the AGC site mildly ascending to 0.0437, while V105G has shown a similar small climb to 0.0430. The differences, fine as they are, could be capable of influencing the kinase binding dynamics or downstream signaling specificity. All the sites in Table 5, above the respective confidence cutoffs, are still theirs to be phosphorylated persistently despite the mutations.

**Table 5:** Predicted phosphorylation scores for ten key sites on the BCL6 protein across the wild type and four mutant variants (I60T, I78T, Q113K, and V105G), based on GPS 6.0 kinase-specific predictions.

Position	Residue	Kinase	Score (WT)	Score (I60T)	Score (I78T)	Score (Q113K)	Score (V105G)	Cutoff
3	S	CK1	0.0815	0.0815	0.0815	0.0815	0.0815	0.0532
16	S	CAMK	0.8841	0.8841	0.8841	0.8841	0.8841	0.0181
92	T	AGC	0.0428	0.0428	0.0420	0.0437	0.0430	0.0197
92	T	CK1	0.0950	0.0950	0.0992	0.0857	0.0939	0.0532
194	S	CK1	0.0668	0.0668	0.0668	0.0668	0.0668	0.0532
271	S	CK1	0.1788	0.1788	0.1788	0.1788	0.1788	0.0532
300	S	CK1	0.0905	0.0905	0.0905	0.0905	0.0905	0.0532
337	S	CK1	0.1449	0.1449	0.1449	0.1449	0.1449	0.0532
345	T	CK1	0.1015	0.1015	0.1015	0.1015	0.1015	0.0532
350	S	CK1	0.0600	0.0600	0.0600	0.0600	0.0600	0.0532

In the NetPhos 3.1 results, wild-type BCL6 shows three very strong phosphorylation predictions at Ser307, Ser308, and Ser366 (all scores ≥0.997). The I60T, Q113K, and V105G mutants keep those same three top sites unchanged, meaning those mutations don't significantly alter BCL6's main phosphorylation hotspots. Only the I78T variant stands out: it creates a new predicted CKII site at Thr78 (score 0.600) that isn't present in the wild type, suggesting I78T could gain an extra regulatory phosphorylation there. Methylation sites were examined using the GPS-MSP 1.0 tool. Using a low threshold, no methylation sites were found in the protein and its mutations. Across wild-type BCL6 and all four point mutants (I60T, V105G, I78T, Q113K), RUBI web server predicts a single high-confidence ubiquitination site despite Q113K introducing an extra lysine—this leaves the total ubiquitylated count unchanged at one, but drops the overall ubiquitylation percentage from 2.63% to 2.56% in the Q113K variant. Meanwhile, all five sequences share an identical 19.83% disorder prediction, and none of the mutated residues lie at the predicted ubiquitination site or shift any region from ordered to disordered (Table 6).

**Table 6:** RUBI web server results.

Variant	Total aa	Lysines	Ubiquitylated Lysines	% Ubiquitylated	% Disorder
**Wild-type**	706	38	1	2.63%	19.83%
**I60T**	706	38	1	2.63%	19.83%
**V105G**	706	38	1	2.63%	19.83%
**I78T**	706	38	1	2.63%	19.83%
**Q113K**	706	39	1	2.56%	19.83%

The wild-type BCL6, as per the NetOGlyc-4.0 web server prediction tool, is believed to have 44 O-glycosylation sites; the Q113K mutant removes that glycan from position 343 (thus a total of 41) but does not introduce any new sites. On the other hand, V105G is losing the site 343 and at the same time, is creating four new sites at 260, 276, 460, and 542—thus, the number becomes 46. The I78T variant removes sites 343 and 455 (making 43) and gains a new site at 260, whereas I60T loses 343 and 455 but adds four new sites at 199, 205, 260, and 276, with a total of 46. The data of these BCL6 NetOGlyc-4.0 web server outcomes declare distinctively, that the change in each case redistributes the glycosylation properties differently across the BCL6 protein and potentially changes its structural flexibility and binding interfaces (Table 7).

**Table 7:** NetOGlyc-4.0 predictions for wild-type BCL6 and each variant.

Variant	Total predicted sites	Lost site(s)	Gained site(s)
**Wild-type**	44	–	–
**Q113K**	41	343	–
**V105G**	46	343	260, 276, 460, 542
**I78T**	43	343, 455	260
**I60T**	46	343, 455	199, 205, 260, 276

PTM predictions showed little difference between wild-type and mutants, although none of the selected nsSNPs appeared to create or delete high-confidence, critical phosphorylation or methylation sites in key functional domains. Small changes in phosphorylation scores at residues such as Thr92 (particularly at I78T and Q113K) could modulate kinase binding affinity or signaling output. Also, minor changes in glycosylation or ubiquitination patterns, such as increased O-glycosylation sites at I60T and V105G, could affect protein solubility, nuclear translocation, or proteasomal degradation rates. Although these findings were predicted in silico and computationally, they indicate that specific mutations have the ability to affect post-translational regulation of BCL6, which could affect its half-life, subcellular localization, or ability to form repressor complexes under physiological conditions.

### 3.10 Gene-gene and domain interaction analyses

**Fig. 10:**
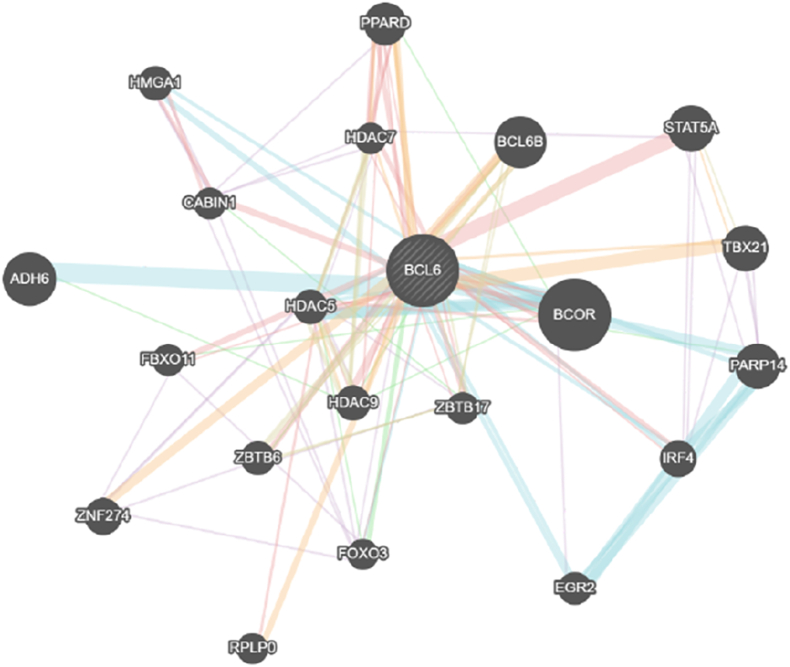
GeneMANIA server BCL6 gene interactions.

**Fig. 11:**
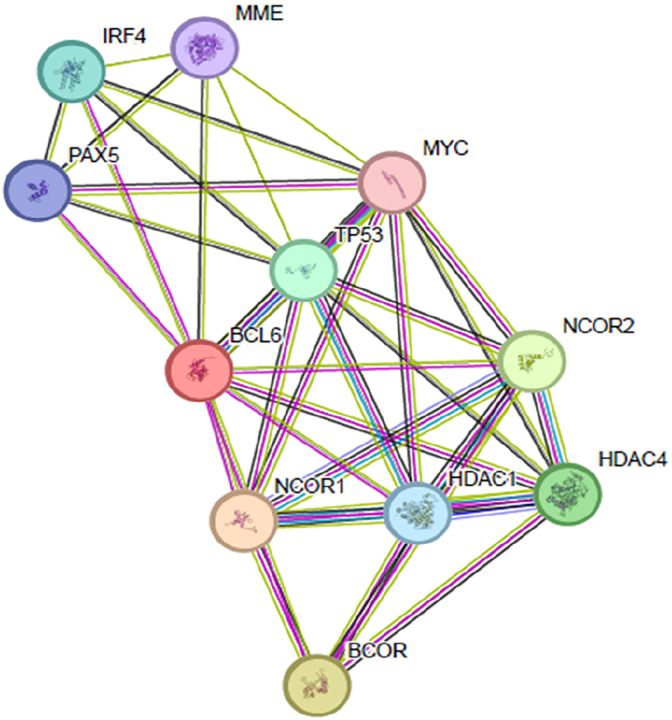
STRING server BCL6 gene interactions.

The GeneMANIA (Fig. 10) and STRING (Fig. 11) web servers were used to experiment with the BCL6 gene's interactions and its interaction network, using the interaction network of this gene with other genes in the obtained interaction network. If the mutation occurs in this gene, the role of other genes in the obtained interaction network may be changed.

The results shown by STRING and GeneMANIA identify a core network of proteins that functionally interact with BCL6. Given that all four selected mutations are in the BTB domain, which plays a critical role in corepressor binding, disruption of this domain may impair BCL6's ability to bind to repressors, which could lead to problems with apoptosis, inflammation, or differentiation. This suggests that disruption of the BTB domain may affect the proper function of B cells.

The Pfam results for both the mutant and wild-type (WT) BCL6 proteins would illuminate the typing of the domain and projected protein characteristics. Both the MUT and WILD sequences confirm the existence of the BTB/POZ domain, which is usually an essential feature of BCL6 related to protein-protein interactions and transcriptional repression as a part. The two sequences also contain the Zinc Finger domains (C2H2 type), which are important for DNA binding and transcription factor activity.

## 4 Discussion

Given the central role of BCL6 in oncogenesis, particularly in diffuse large B-cell lymphomas (DLBCL), understanding the functional consequences of nsSNPs in the *BCL6* gene and their structural effects on BCL6 protein seems crucial (Pasqualucci et al., 2003a, 2003b; Ebid et al., 2012). Herein, nsSNPs of BCL6 were analyzed and selected based on high-confidence deleteriousness scores. The selected nsSNPs were then subjected to structural modeling, docking with key BCL6 corepressors (including BCOR, NCOR, and SMRT), and MD simulations of BCL6-corepressor complexes. The obtained results were used to explore the effects of nsSNPs on the structure and function of the BCL6 protein. In this regard, 54 nsSNPs were identified as potentially deleterious based on consensus predictions performed by Meta-SNP, SIFT, PolyPhen-2, PhD-SNP, and SNAP tools (out of 577 nsSNPs initially retrieved from databases). These tools consistently inferred that these mutations can affect BCL6 function. Among 54 nsSNPs, the Q113K, V105G, I78T, and I60T mutations within the BTB domain showed the highest potential to have functional consequences. These mutation sites are located within or near BLC6 functional motifs, which are implicated in corepressor binding (Melnick et al., 2002). Previously, it has been demonstrated that 3 mutations (N21K, S59A, and H116A) in the BTB domain can impair the functions of BCL6 (Ahmad et al., 2003). These mutations could serve as a control for our procedure to validate the obtained results. They have reported that the H116A mutation has a significantly reduced affinity for Trx-(SMRT-BBD), while the N21K mutation abrogated the interaction. In contrast to wild-type BCL6 BTB, the N21K, H116A, or N21K/H116A mutants were unable to repress transcription. However, these same mutations in full-length BCL6 resulted in greatly reduced repression activity, but not abrogation. Hence, it was deduced that motifs in other regions of BCL6 could compensate for the defect in BTB corepressor recruitment. The lateral groove of the BCL6 BTB domain is required for both physical and functional interaction between BCL6-and BBD-containing corepressors. In another study, Ser 59 was reported to be important for the SCF FBXL17-dependent degradation of a BCL6 BTB domain. In vitro assay showed that mutation of Ser 59 in BCL6 interfered with ubiquitination by the SCF(FBXL17) complex (Mena et al., 2018). They suggested that S59G and H116R variations affect the BCL6 interactions and functions.

Based on the predictions of VEP and ANNOVAR tools, a plausible link can exist between these mutations and the altered repressive activity of the BCL6. Initial structural analyses using the I-Mutant tool revealed that selected 4 nsSNPs were associated with significant changes in protein stability. Since these SNPs primarily reside within the BTB domain, SNP-mediated changes in structural stability of BCL6 would change its dimerization and corepressor recruitment (Lin et al., 2018). It should be noted that in both I60T and I78T substitutions, the larger, non-polar Isoleucine is changed to a smaller, polar Threonine. Although these positions are still hidden inside the protein, they no longer appear strongly positively charged. In contrast, they become negative in terms of their hydrophobicity scores. This could lead to a slightly more loosely packed region in the BCL6 protein. The V105G mutation is a direct replacement of valine with glycine. This mutation involves a residue that is buried in the protein's core. Glycine's small side chain allows for greater rotational freedom, which could lead to higher flexibility in the protein core of BCL6 (Khan et al., 2007). The Q113K mutation replaces a neutral molecule, glutamine, with a positively charged lysine. The presence of positively charged residues can enhance the electrostatic interactions, which are essential for protein stability and function (Sokalingam et al., 2012). Moreover, this change in the local charge of the protein can strengthen the protein binding with negatively charged partners and potentially improve the functional interactions of the protein (Wu et al., 2010).

Lack of changes in the disulfide bonds and maintaining the original secondary structures in the mutant forms indicated that the exerted mutation-derived structural realignments do not make a drastic change in the structural integrity of BCL6. This fact could be followed in the drawn phylogenetic tree, in which the V105G seems to be the most divergent mutation due to the introduction of a new glycine residue. Moreover, the introduction of these mutations does not lead to significant changes in post-translational modifications, interaction network, gene expression, and domain annotation. Protein-protein docking results showed a significant decrease in the binding affinity of mutated BCL6 structures. It should be noted that the V105G and Q113K mutants showed the highest reduction in binding affinity against the BCOR peptide. This was anticipated due to the structural changes triggered by the mutated amino acids. The 2D interaction plots also showed that the binding residues are misaligned in the mutated BCL6 complexes. This misalignment could be rooted in the local structural distortions introduced by the mutations. In the case of NCOR, the V105G mutant might be associated with the displacement of the co-repressor recognition helix. This structural realignment can reduce hydrophobic and polar contacts, which are necessary for high-affinity BCL6 interactions. The binding specificity of the BTB domain is highly dependent on the physical interactions and the electrostatic environment of the interaction site (Perez‐Torrado et al., 2006). It has also been shown that significant changes occurred in the charge distribution of the mutated complexes. Positively charged regions of the BTB domain are important for its intact function. These regions attract the negatively charged corepressor residues (Stogios et al., 2005). Therefore, their neutralization could contribute to the impaired binding observed in our docking studies (Melnick et al., 2002). Since these electrostatic disturbances can extend beyond direct interaction zones, a broader allosteric effect could be assumed on the conformation and binding capabilities of the BTB domain. In light of these observations, it could be concluded that even single-residue changes within the BTB domain can disrupt the molecular interactions, which are required for co-repressor recognition. Loss of such interactions would directly reduce the ability of BCL6 to repress its target genes. Dysregulation of BCL6 function could deflect the proliferation pathways and immune signaling (Huang et al., 2013; Lundell-Smith, 2017).

MD simulations were employed to screen the dynamic behavior of the mutants under physiological conditions. Compared to wild-type BCL6, the mutants' BTB domains exhibited higher RMSD values. The RMSD plots indicated that structural instability of mutant structures has increased over time. On the other hand, RMSF profiles showed increased flexibility in loop regions, which are close to the dimer and co-repressor binding interfaces. These profiles can potentially affect the molecular recognition (Mukherjee et al., 2024; Mansouri et al., 2013). In the Q113K mutant, the fluctuation in the binding helix was nearly doubled compared to the wild type. This could be construed as the loss of structural rigidity (Rader, 2002; Rader et al., 2002), which is needed for precise docking. Furthermore, analysis of Rg plots indicated a slight compaction in the structure of the mutant forms. This possibly could indicate a partial collapse of the hydrophobic core. However, since elevated structural compactness was not associated with an increased structural order, it could imply misfolding of the BTB domain or formation of its aggregation-prone intermediates. Misfolded proteins often form aggregates, as seen in γD-crystallin mutations linked to congenital cataracts, which disrupt structural stability and solubility (Lin et al., 2024). Maintaining the conserved hydrogen bond networks during the MD simulation is an essential factor in structural and functional integrity (Shabaev et al., 2022; Thayer et al., 2016). In this regard, MD-based hydrogen bond analyses demonstrated that some of the key hydrogen bonds are disrupted in the mutants. Altered hydrogen bonds were most notable in the V105G and Q113K mutants, where disruption of H-bonds led to local unfolding of the adjacent α-helix. Formation of new hydrogen bonds in mutants can lead to conformational changes (Imam et al., 2025). These changes can, in turn, alter the docking site topology, lead to disturbed communication between functional sites, and consequently can lead to ineffective transmission of conformational signals required for transcriptional repression.

The obtained results demonstrated that the analyzed SNPs could deregulate the BCL6-mediated gene repression in lymphoid cells. This effect could be more obvious in mutants that can impair corepressor binding. The inability of BCL6 to recruit its corepressors may lead to continuous expression of genes involved in proliferation or immune evasion, which could eventually lead to oncogenesis (Liongue et al., 2024; McLachlan et al., 2022). Moreover, the altered structural behavior of the mutated BCL6 molecules during MD simulations could indicate their higher susceptibility to proteasomal degradation or abnormal post-translational modifications. These unwanted consequences could result in more complicated deleterious effects of mutated BCL6 proteins. On the other hand, the selected SNPs could serve as potential biomarkers to identify patients at risk of BCL6-driven malignancies or those that are resistant to therapies targeting this pathway (Karimi et al., 2022). Understanding the conformational consequences of specific mutations can be helpful in the design of small molecules. These drugs can either stabilize the mutant form or restore its ability to interact with corepressors. In particular, the structural changes identified in this study can shed light on rational drug design, which targets the BTB domain interface. Reduced structural stability and increased flexibility in key regions of the BTB domain are evident in mutants, especially Q113K and V105G, due to increased RMSD and RMSF values. These may disrupt corepressor interaction surfaces or improper domain folding, which can prevent the formation of repressor complexes. Functional impairment is also evident in some mutants according to radius of gyration (Rg) plots due to loss of compactness.

This research is restricted by the fact that only computational tools have been used. Even though these tools offer some insights into the structural and functional consequences of BCL6 nsSNPs, they are not able to replace experimental verification completely. We recommend that upcoming investigations carry out site-directed mutagenesis of certain SNPs together with co-immunoprecipitation to check if corepressor binding is possible, luciferase assays for testing transcriptional repression, and western blotting or mass spectrometry for the detection of stability and post-translational modifications. Such experimental verification will be indispensable for transferring these predictions from the lab to the real world and to clinical settings.

## 5 Conclusion

The present in silico investigation showed that selected SNPs in the BTB domain of BCL6 can significantly affect its structural integrity, ability for dimerization, and capacity for interaction with corepressor molecules. The docking and MD simulation data showed structural destabilization, interaction loss, and potential impaired function of mutated BCL6 molecules. Although the obtained results can shed light on the effects of SNPs on the BCL6 function, future studies should be carried out for their experimental validation. Methods such as mutagenesis, co-immunoprecipitation, and transcriptional assays would be most amenable to confirm the computational findings.

## 6 Limitations and future work

The present study is an in silico study based on predictions and simulations. Although we have used a multi-tool consensus approach, employed the most recent and validated tools and extended simulation times (with replicates), and validated the predictions against the crystal structures of the BCL6 BTB domain and its corepressors, experimental validation of the obtained results remains essential to confirm the functional impact of the selected nsSNPs. Resolving the full-length structure of BCL6, site-directed mutagenesis of the BCL6 BTB domain according to the selected nsSNPs, co-immunoprecipitation assays to assess corepressor binding affinity, luciferase reporter assays to measure transcriptional repression activity, and Western blotting or mass spectrometry to evaluate protein stability and PTMs could be prioritized as future work of this study.

## Ethics approval and consent to participate

Not applicable.

## Consent for publication

Not applicable.

## Availability of data and materials

The employed data is clearly addressed within the study.

## Credit author statement

Conceptualization: Shaden M. H. Mubarak, Pardis Abdali Dehdezi, Amir Razavinia, Bahman Khalesi, Zahra Sadat Hashemi, Abolfazl Jahangiri, Mohammad Reza Rahbar, Mahdieh Mahboobi, Saeed Khalili; Methodology: Shaden M. H. Mubarak, Pardis Abdali Dehdezi, Amir Razavinia, Bahman Khalesi, Zahra Sadat Hashemi, Abolfazl Jahangiri, Mohammad Reza Rahbar, Mahdieh Mahboobi, Saeed Khalili; Investigation: Shaden M. H. Mubarak, Pardis Abdali Dehdezi, Amir Razavinia, Bahman Khalesi, Zahra Sadat Hashemi, Abolfazl Jahangiri, Mohammad Reza Rahbar, Mahdieh Mahboobi, Saeed Khalili; Formal analyses: Shaden M. H. Mubarak, Pardis Abdali Dehdezi, Amir Razavinia, Bahman Khalesi, Zahra Sadat Hashemi, Abolfazl Jahangiri, Mohammad Reza Rahbar, Mahdieh Mahboobi, Saeed Khalili.; Writing - Original Draft: Shaden M. H. Mubarak, Pardis Abdali Dehdezi, Amir Razavinia, Bahman Khalesi, Zahra Sadat Hashemi, Abolfazl Jahangiri, Mohammad Reza Rahbar, Mahdieh Mahboobi, Saeed Khalili; Writing - Review & Editing: Shaden M. H. Mubarak, Pardis Abdali Dehdezi, Amir Razavinia, Bahman Khalesi, Zahra Sadat Hashemi, Abolfazl Jahangiri, Mohammad Reza Rahbar, Mahdieh Mahboobi, Saeed Khalili; Supervision: Saeed Khalili.

## Declaration of AI use

The authors confirm that no generative AI tools were used in the design, execution, or interpretation of this study. All experimental data, statistical analyses, and manuscript preparation were conducted through traditional human-led methods. AI-based software was employed only for routine tasks such as language editing and creating schematic illustrations (unrelated to experimental data), and all outputs were critically reviewed and validated by the authors for accuracy and clarity.

## Funding

This research did not receive any specific grant from funding agencies in the public, commercial, or not-for-profit sectors.

## Declaration of competing interest

The authors declare that they have no known competing financial interests or personal relationships that could have appeared to influence the work reported in this paper.

## References

[bib1] Ahmad K.F. (2003). Mechanism of SMRT corepressor recruitment by the BCL6 BTB domain. Mol. Cell.

[bib2] Basso K., Dalla-Favera R. (2010). BCL6: master regulator of the germinal center reaction and key oncogene in B cell lymphomagenesis. Adv. Immunol..

[bib3] Benkert P., Künzli M., Schwede T. (2009). QMEAN server for protein model quality estimation. Nucleic Acids Res..

[bib4] Bonchuk A., Balagurov K., Georgiev P. (2023). BTB domains: a structural view of evolution, multimerization, and protein–protein interactions. Bioessays.

[bib5] Boyle A.P. (2012). Annotation of functional variation in personal genomes using RegulomeDB. Genome Res..

[bib6] Buel G.R., Walters K.J. (2022). Can AlphaFold2 predict the impact of missense mutations on structure?. Nat. Struct. Mol. Biol..

[bib7] Calabrese R., Capriotti E., Casadio R. (2008). EMBNET08.

[bib8] Capriotti E., Fariselli P., Casadio R. (2005). I-Mutant2. 0: predicting stability changes upon mutation from the protein sequence or structure. Nucleic Acids Res..

[bib9] Capriotti E., Altman R.B., Bromberg Y. (2013). Collective judgment predicts disease-associated single nucleotide variants. BMC Genom..

[bib10] Cardenas M.G. (2017). The expanding role of the BCL6 oncoprotein as a cancer therapeutic target. Clin. Cancer Res..

[bib11] Das R., Upadhyai P. (2017). Application of geographic population structure (GPS) algorithm for biogeographical analyses of populations with complex ancestries: a case study of south Asians from 1000 genomes project. BMC Genet..

[bib12] Ebid G.T. (2012). Genetic polymorphism study of the BCL-6 gene, in diffuse large B-cell lymphoma. Saudi J. Health Sci..

[bib13] Finn R.D. (2006). Pfam: clans, web tools and services. Nucleic Acids Res..

[bib14] Garg V.K. (2016). MFPPI–multi FASTA ProtParam interface. Bioinformation.

[bib15] Honorato R.V. (2024). The HADDOCK2. 4 web server for integrative modeling of biomolecular complexes. Nat. Protoc..

[bib16] Hossain M.S., Roy A.S., Islam M.S. (2020). In silico analysis predicting effects of deleterious SNPs of human RASSF5 gene on its structure and functions. Sci. Rep..

[bib17] Huang C., Hatzi K., Melnick A. (2013). Lineage-specific functions of Bcl-6 in immunity and inflammation are mediated by distinct biochemical mechanisms. Nat. Immunol..

[bib18] Imam I.A. (2025). L858R/L718Q and L858R/L792H mutations of EGFR inducing resistance against osimertinib by forming additional hydrogen bonds. Proteins: Struct., Funct., Bioinf..

[bib19] Iqbal M.W. (2024). Analysis of damaging non-synonymous SNPs in GPx1 gene associated with the progression of diverse cancers through a comprehensive in silico approach. Sci. Rep..

[bib66] Jahangiri A. (2012). Precise detection of *L. monocytogenes* hitting its highly conserved region possessing several specific antibody binding sites. J. Theor. Biol..

[bib20] Karimi S. (2022). Impact of SNPs, off-targets, and passive permeability on efficacy of BCL6 degrading drugs assigned by virtual screening and 3D-QSAR approach. Sci. Rep..

[bib64] Khalili S. (2017). In silico prediction and in vitro verification of a novel multi-epitope antigen for HBV detection. Mol. Genet. Microbiol. Virol..

[bib21] Khan S., Vihinen M. (2007). Spectrum of disease-causing mutations in protein secondary structures. BMC Struct. Biol..

[bib22] Khrustalev V.V., Barkovsky E.V. (2012). In silico directed mutagenesis using software for glycosylation sites prediction as a new step in antigen design. Journal of Integrated OMICS.

[bib23] Kumar P., Henikoff S., Ng P.C. (2009). Predicting the effects of coding non-synonymous variants on protein function using the SIFT algorithm. Nat. Protoc..

[bib24] Kusumaningrum S. (2014). The molecular docking of 1, 4-naphthoquinone derivatives as inhibitors of Polo-like kinase 1 using Molegro Virtual Docker. J. Appl. Pharmaceut. Sci..

[bib25] Landrum M.J. (2016). ClinVar: public archive of interpretations of clinically relevant variants. Nucleic Acids Res..

[bib26] Li S. (2007). Snap: an integrated SNP annotation platform. Nucleic Acids Res..

[bib27] Li S. (2021). pCysMod: prediction of multiple cysteine modifications based on deep learning framework. Front. Cell Dev. Biol..

[bib28] Lin L.-Y. (2018). Backbone resonance assignment of the BCL6-BTB/POZ domain. Biomolecular NMR Assignments.

[bib29] Lin N. (2024). Truncation mutations of CRYGD gene in congenital cataracts cause protein aggregation by disrupting the structural stability of γD-crystallin. Int. J. Biol. Macromol..

[bib30] Liongue C., Almohaisen F.L., Ward A.C. (2024). B cell lymphoma 6 (BCL6): a conserved regulator of immunity and beyond. Int. J. Mol. Sci..

[bib31] Lundell-Smith G.G. (2017).

[bib32] MacArthur J. (2017). The new NHGRI-EBI Catalog of published genome-wide association studies (GWAS Catalog). Nucleic Acids Res..

[bib33] Manfredi M. (2021). DeepREx-WS: a web server for characterising protein–solvent interaction starting from sequence. Comput. Struct. Biotechnol. J..

[bib34] Mansouri S. (2013). Molecular dynamic study of human prion protein upon D178N mutation: new perspective to H-bonds, salt bridges and the critical amino acids. Protein Pept. Lett..

[bib35] Matvienko M. (2015). *CLC genomics workbench,* plant anim. Genome Sr. Field. Appl. Sci. CLC Bio..

[bib36] McGuffin L.J., Bryson K., Jones D.T. (2000). The PSIPRED protein structure prediction server. Bioinformatics.

[bib37] McLachlan T. (2022). B-cell lymphoma 6 (BCL6): from master regulator of humoral immunity to oncogenic driver in pediatric cancers. Mol. Cancer Res..

[bib38] McLaren W. (2016). The ensembl variant effect predictor. Genome Biol..

[bib39] Melnick A. (2002). Critical residues within the BTB domain of PLZF and Bcl-6 modulate interaction with corepressors. Mol. Cell Biol..

[bib40] Mena E.L. (2018). Dimerization quality control ensures neuronal development and survival. Science.

[bib65] Mohammadpour H., Khalili S., Hashemi Z.S. (2015). Kremen is beyond a subsidiary co-receptor of Wnt signaling: an in silico validation. Turk. J. Biol..

[bib41] Mukherjee S., Mukherjee M., Mishra P. (2024).

[bib42] Pasqualucci L. (2003). Transcriptional deregulation of mutated BCL6 alleles by loss of negative autoregulation in diffuse large B cell lymphoma. Ann. N. Y. Acad. Sci..

[bib43] Pasqualucci L. (2003). *Mutations of the BCL6 proto-oncogene disrupt its negative autoregulation in diffuse large B-cell lymphoma,* Blood. The Journal of the American Society of Hematology.

[bib44] Paz J.O., Batchelor W.D., Pedersen P. (2004). WebGro: a web‐based soybean management decision support system. Agron. J..

[bib45] Perez‐Torrado R., Yamada D., Defossez P.A. (2006). Born to bind: the BTB protein–protein interaction domain. Bioessays.

[bib46] Rader A.J. (2002).

[bib47] Rader A. (2002). Protein unfolding: rigidity lost. Proc. Natl. Acad. Sci..

[bib48] Shabaev A. (2022). Infrared Technology and Applications XLVIII.

[bib49] Sivakumar K., Sidhic N., Subbiah U. (2025). In silico prediction of non-synonymous SNPs in the human CALCR gene. Curr. Pharmacogenomics Personalized Med. (CPPM).

[bib50] Snel B. (2000). STRING: a web-server to retrieve and display the repeatedly occurring neighbourhood of a gene. Nucleic Acids Res..

[bib51] Sokalingam S. (2012). A study on the effect of surface lysine to arginine mutagenesis on protein stability and structure using green fluorescent protein. PLoS One.

[bib52] Stanfill A.G., Cao X. (2021). Enhancing research through the use of the genotype-tissue expression (GTEx) database. Biol. Res. Nurs..

[bib53] Stogios P.J. (2005). Sequence and structural analysis of BTB domain proteins. Genome Biol..

[bib54] Thayer K.M., Quinn T.R. (2016). p53 R175H hydrophobic patch and h‐bond reorganization observed by MD simulation. Biopolymers.

[bib55] Wang K., Li M., Hakonarson H. (2010). ANNOVAR: functional annotation of genetic variants from high-throughput sequencing data. Nucleic Acids Res..

[bib56] Wang M. (2013). Recent advances in predicting functional impact of single amino acid polymorphisms: a review of useful features, computational methods and available tools. Curr. Bioinf..

[bib57] Wu X.P., Li X.K. (2010). Effect of charge at an amino acid of basic fibroblast growth factor on its mitogenic activity. Chin. Chem. Lett..

[bib58] Xue L.C. (2016). PRODIGY: a web server for predicting the binding affinity of protein–protein complexes. Bioinformatics.

[bib59] Yates C.M., Sternberg M.J. (2013). The effects of non-synonymous single nucleotide polymorphisms (nsSNPs) on protein–protein interactions. J. Mol. Biol..

[bib60] Yazar M., Özbek P. (2021). In silico tools and approaches for the prediction of functional and structural effects of single-nucleotide polymorphisms on proteins: an expert review. OMICS A J. Integr. Biol..

[bib61] Zhang Y., Skolnick J. (2005). TM-align: a protein structure alignment algorithm based on the TM-score. Nucleic Acids Res..

[bib62] Zhou X.A. (2018). Genomic analyses identify recurrent alterations in immune evasion genes in diffuse large B-cell lymphoma, leg type. J. Invest. Dermatol..

[bib63] Zuberi K. (2013). GeneMANIA prediction server 2013 update. Nucleic Acids Res..

