# Supplementary material for: Decoding the impact of nsSNP variants on BCL6 function through integrated computational analysis
Source: Curr Res Struct Biol. 2026 Feb 2;11:100180. doi: 10.1016/j.crstbi.2026.100180 (PMC12917388; doi:10.1016/j.crstbi.2026.100180)
Supplement: Multimedia component 1 [file mmc1.docx]

|  | **1** | **2** | **3** | **4** | **5** | **6** | **7** | **8** | **9** | **10** | **11** | **12** | **13** | **14** | **15** |
| --- | --- | --- | --- | --- | --- | --- | --- | --- | --- | --- | --- | --- | --- | --- | --- |
|  | Wild-type BCL 6 and SMRT | I60T mutant and SMRT | I78T mutant and SMRT | V105G mutant and SMRT | Q113K mutant and SMRT | Wild-type BCL6 and NCoR | I60T mutant and NCoR | I78T mutant and NCoR | V105G mutant and NCoR | Q113K mutant and NCoR | Wild-type BCL6 and BCoR | I60T mutant and BCoR | I78T mutant and BCoR | V105G mutant and BCoR | Q113K and BCoR |
| ΔG (kcal/mol) | -8.6 | -7.9 | -7.5 | -8.7 | -8.5 | -8.6 | -8.5 | -8.4 | -6.9 | -8.1 | -10.6 | -7.3 | -7.8 | -6.3 | -7.0 |
| HADDOCK score | -91.1 +/- 1.8 | -86.0 +/- 5.9 | -85.1 +/- 2.0 | -93.0 +/- 3.7 | -86.2 +/- 1.1 | -72.5 +/- 1.6 | -76.4 +/- 2.2 | -83.8 +/- 7.1 | -76.0 +/- 1.3 | -75.2 +/- 3.1 | -79.5 +/- 8.4 | -73.6 +/- 6.2 | -73.4 +/- 4.3 | -84.5 +/- 5.7 | -59.0 +/- 5.5 |
| Cluster size | 13 | 35 | 32 | 25 | 57 | 93 | 61 | 52 | 50 | 28 | 6 | 6 | 74 | 6 | 23 |
| RMSD from the overall lowest-energy structure | 10.0 +/- 0.1 | 0.5 +/- 0.3 | 10.5 +/- 0.1 | 10.6 +/- 0.4 | 10.6 +/- 0.0 | 1.6 +/- 0.3 | 1.7 +/- 0.1 | 0.7 +/- 0.5 | 3.0 +/- 0.3 | 7.6 +/- 0.1 | 6.8 +/- 0.1 | 8.1 +/- 0.3 | 9.5 +/- 0.3 | 9.3 +/- 0.1 | 5.9 +/- 0.2 |
| Van der Waals energy | -44.8 +/- 4.0 | -40.9 +/- 2.9 | -45.2 +/- 6.4 | -41.5 +/- 4.0 | -55.3 +/- 1.9 | -36.5 +/- 6.5 | -37.6 +/- 1.9 | -43.8 +/- 6.1 | -38.4 +/- 2.7 | -46.1 +/- 1.2 | -55.6 +/- 4.0 | -42.5 +/- 3.5 | -46.3 +/- 3.1 | -43.6 +/- 1.5 | -34.0 +/- 3.9 |
| Electrostatic energy | -123.1 +/- 10.1 | -206.2 +/- 30.2 | -165.3 +/- 44.2 | -235.3 +/- 17.7 | -114.4 +/- 11.4 | -77.0 +/- 24.5 | -87.2 +/- 10.8 | -86.3 +/- 8.7 | -75.5 +/- 6.7 | -93.7 +/- 14.1 | -72.9 +/- 28.4 | -15.6 +/- 6.2 | -25.2 +/- 4.2 | -117.7 +/- 36.8 | -17.4 +/- 8.1 |
| Desolvation energy | -24.7 +/- 0.7 | -6.0 +/- 1.8 | -9.4 +/- 2.2 | -6.8 +/- 2.4 | -9.1 +/- 2.5 | -20.9 +/- 1.8 | -22.0 +/- 2.2 | -23.1 +/- 1.0 | -22.8 +/- 2.0 | -12.9 +/- 2.1 | -12.1 +/- 4.6 | -29.9 +/- 1.5 | -22.8 +/- 1.7 | -18.2 +/- 2.3 | -22.1 +/- 0.5 |
| Restraints violation energy | 30.6 +/- 22.2 | 20.5 +/- 14.2 | 26.7 +/- 17.9 | 23.9 +/- 13.4 | 11.1 +/- 3.5 | 2.4 +/- 0.5 | 6.5 +/- 3.1 | 3.6 +/- 2.3 | 2.6 +/- 1.4 | 25.9 +/- 21.4 | 28.6 +/- 16.1 | 19.0 +/- 7.9 | 8.1 +/- 11.4 | 8.5 +/- 0.9 | 5.6 +/- 3.0 |
| Buried Surface Area | 1444.0 +/- 43.9 | 1522.6 +/- 41.4 | 1532.2 +/- 47.8 | 1519.4 +/- 85.1 | 1458.4 +/- 24.0 | 1121.1 +/- 147.6 | 1285.8 +/- 59.3 | 1364.4 +/- 83.7 | 1234.6 +/- 52.5 | 1353.1 +/- 110.8 | 1455.2 +/- 55.8 | 1119.4 +/- 92.3 | 1089.6 +/- 21.6 | 1493.9 +/- 100.2 | 991.9 +/- 8.1 |
| Z-Score | -1.6 | -1.4 | -2.3 | -1.9 | -1.7 | -1.4 | -1.6 | -1.8 | -1.1 | -1.4 | -2.3 | -1.2 | -1.9 | -1.2 | -1.1 |

Supplementary Table: Docking and binding affinity results of BCL6 and its mutant proteins on the co-repressors (BCoR, NCoR, and SMRT). The Wild-type ΔG was calculated by re-docking of BCL6 with its wild type BCoR, NCoR, and SMRT co-repressors (BCL6-BCoR complex was obtained from PDB ID: 3BIM; BCL6-NCoR complex was obtained from PDB ID: 6XYX; BCL6-SMRT complex was obtained from PDB ID: 1R2B).
